# Mapping Adolescent Wellbeing: Developmental Network Differences between Early To Middle Adolescence in 24 Countries

**DOI:** 10.1007/s10964-026-02330-z

**Published:** 2026-02-17

**Authors:** Wanying Zhou, Jose Marquez, Leoni Boyle, Laura Taylor

**Affiliations:** 1 Wellbeing Research Centre, University of Oxford, Oxford, UK; 2 Manchester Institute of Education, University of Manchester, Manchester, UK

**Keywords:** Adolescent wellbeing, Psychometric network analysis, Developmental differences, Cross-culture, Future-orientation

## Abstract

Adolescent wellbeing is often assessed using composite scores, yet less is known about how specific components of wellbeing are structurally organized and how this organization shifts across development. Using psychometric network analysis, we examined interconnections among 49 wellbeing indicators spanning subjective wellbeing (life satisfaction, affect, and domain satisfaction) and psychological wellbeing (flourishing and positive mental functioning) in a large international sample of adolescents. Data were drawn from 6,445 students aged 11–18 years (M = 14.4, SD = 1.96; 51.7% girls; 3.1% preferred not to report gender) recruited from 38 schools across 24 countries. Networks were estimated for the full sample and separately for early adolescence (11–14 years; 51.9%) and middle adolescence (15–18 years; 48.1%). Across all analyses, overall life satisfaction and satisfaction with student life consistently emerged as the most central nodes, underscoring their integrative role in adolescents’ wellbeing evaluations. Network density was similar across age groups, indicating comparable overall interconnectedness; however, network configuration differed developmentally. In middle adolescence, future-oriented optimism became more structurally prominent, whereas present-focused life evaluation (current life satisfaction) showed reduced centrality. Indicators reflecting negative affect and calmness also showed modest age-related increases in relative importance. Together, these findings suggest a developmental reorganization of adolescent wellbeing from present-oriented evaluations toward future-oriented expectations and regulatory resources, while reaffirming the central role of overall life satisfaction and student-life satisfaction. Mapping age-related differences in wellbeing structure across a large cross-national sample informs age-sensitive approaches to assessment, monitoring, and intervention.

## Introduction

There is growing interest in promoting the wellbeing of children and adolescents both within educational settings and more broadly (Marquez et al., 2024; OECD, 2021; Taylor et al., 2022). This increased attention is driven by several factors. First, a number of countries have reported declining trends in adolescent wellbeing over the past 15 to 20 years, with younger cohorts consistently reporting lower levels of wellbeing over time (Marquez et al., 2024). Second, adolescence represents a critical developmental window during which wellbeing tends to decline, with decreases often beginning around age 11 (Casas & Gonzalez-Carrasco, 2019). Mental health difficulties also frequently emerge during this period, with the average onset age around 14.5 years (Solmi et al., 2022). Third, higher adolescent wellbeing has been robustly linked to a wide range of positive life outcomes, including academic achievement, physical and mental health, social relationships (Geijsen & Bartels, 2024) and labour market success (De Neve & Oswald, 2012). Importantly, researchers have argued there is no trade-off between academic and wellbeing outcomes, supporting the case for prioritising student wellbeing in schools (Clarke, 2020), including contexts such as England (Putwain et al., 2020), China (Zhou & McLellan,^ 2021^), Sweden (Bortes et al., 2021), and Australia (Cárdenas et al., 2022). To support these efforts, this study uses psychometric network analysis to map how 49 indicators of wellbeing interrelate in 11 to 18-year-olds, to evaluate network stability, and to test whether the structure differs between early and middle adolescence.

Any effort to promote wellbeing must begin with a clear conceptualisation of what is meant by the term. Wellbeing has traditionally been understood through two main theoretical perspectives. Subjective wellbeing, or hedonic wellbeing, emphasizes feeling good and includes both affective (positive and negative emotions) and cognitive (life satisfaction) components (Diener et al., 2002). In contrast, psychological wellbeing, or eudaimonic wellbeing, focuses on functioning well and is typically defined by constructs such as autonomy, purpose in life, environmental mastery, optimism, personal growth, self-acceptance, and positive relationships (Ryff et al., 2021). Although foundational wellbeing frameworks and constructs were developed for adults (e.g., Diener et al., 1985; Ryff, 1989), these have been adapted and validated for children and adolescents (Casas & González-Carrasco, 2022), with wide consensus that comprehensive youth wellbeing assessment should capture both hedonic and eudaimonic elements (Sarriera & Bedin, 2017).

Efforts to promote wellbeing also require clarity in how these wellbeing dimensions are measured. Traditional measurement approaches have relied on latent variable models, such as factor analysis and structural equation modelling (Diener et al., 1999; Ryff & Keyes, 1995), which posit that wellbeing indicators reflect an underlying construct. From this perspective, subjective wellbeing is a latent factor that accounts for variance in life satisfaction and affect, while psychological wellbeing is seen as a latent dimension explaining constructs such as self-acceptance, autonomy, and purpose. However, recent work has proposed network analysis as a promising alternative to wellbeing measurement (Borsboom, 2017). The network approach conceptualizes psychological phenomena as systems of mutually interacting components, where constructs like psychological wellbeing are understood to emerge from dynamic interactions among their indicators. For example, self-acceptance may reinforce positive relationships, which in turn could foster life purpose collectively giving rise to psychological wellbeing as an emergent property.

Network methods add value by mapping how specific elements (e.g., life satisfaction, affect, purpose) directly associate, detecting redundant or low-uniqueness items to improve measurement efficiency (Christensen et al., 2020), and identifying central nodes that may serve as high-leverage intervention targets (Bringmann et al., 2019; Epskamp et al., 2018). They also enable comparisons across groups (age, gender) and, when longitudinal data are available, can model how changes in one domain propagate through the system (Dalege et al., 2017; Hevey, 2018; Robinaugh et al., 2020). Because network models are not tied to predefined latent constructs, they offer an open, visual framework for refining theory, informing interventions, and guiding policy (Marsman et al., 2017). A key critique, however, is that centrality and edge estimates are conditional on the selected node set and sensitive to measurement overlap: different item pools or covariates can produce different centrality rankings (Bringmann et al., 2019; Epskamp et al., 2018). In addition, recent work re-analysing hundreds of published networks found that a large share of edges in network studies are supported by weak or inconclusive statistical evidence (Huth et al., 2025).

The use of network analysis in the adolescent wellbeing literature remains scarce, but has grown in recent years. Most studies have focused on risk and protective factors for wellbeing (Wang et al., 2024) and related constructs such as internalizing difficulties (Black et al., 2023), as well as in the interconnections between wellbeing and other psychological constructs such as character strengths (Blasco-Belled, 2023), and mental health symptoms (Tejada-Gallardo et al., 2022) including depression and anxiety (Campbell & Osborn, 2021; Wasil et al., 2021).

Only a few studies have applied network analysis to map wellbeing structure, either by jointly examining both major domains (subjective wellbeing and psychological wellbeing) or by comprehensively covering the full range of subjective wellbeing components. For example, in a study of 4,282 Chinese high school students (mean age = 16.32), applied network analysis to items from one scale - the General Well-Being Schedule scale (Wang et al., 2023). While this instrument primarily assesses subjective wellbeing (mostly negative affect), it also includes elements of psychological wellbeing (e.g., vitality, purpose). Their findings showed that the item “Have you been anxious, worried, or upset?” was the most central node, suggesting it may be a strategic target for interventions.

Another study involved 888 Spanish adolescents (ages 12–16), and examined the longitudinal associations between cyberbullying victimisation and subjective wellbeing using psychometric network analysis (Vieta-Piferrer et al., 2024). Participants completed measures of overall and domain-specific life satisfaction as well as positive and negative affect at two time points. Despite including a contextual risk factor (cyberbullying), the study provided valuable insights into the internal structure of subjective wellbeing. Overall life satisfaction emerged as a central node across life domains, and the affect item “happy” showed the highest centrality at both time points. Using the same sample, a study explored the interactions between subjective wellbeing and psychological wellbeing over time and showed that positive affect, particularly feeling happy and satisfied, acted as key connectors between the two domains, while negative affect (e.g., worry) was inversely linked to psychological wellbeing longitudinally (Blasco-Belled et al., 2024). These results point to specific components that may drive change across wellbeing domains. However, neither study examined differences across age groups or used a cross-cultural sample, limiting the ability to draw conclusions about age-group variation and cross-cultural generalisability.

Only one study to date has used network analysis to examine adolescent wellbeing using a multi-cultural sample. In a cross-sectional study of adolescents in India (ages 12–18, *N* = 310), Israel (12–18, *N* = 306), and the United Kingdom (12–25, *N* = 1,666), the Short Warwick-Edinburgh Mental Well-Being Scale (SWEMWBS) was employed to assess wellbeing during the COVID-19 pandemic (Shukla et al., 2022). This scale primarily reflects psychological wellbeing (functioning, agency, connectedness) and affect (relaxed, useful, optimistic). Network analysis revealed cultural variation in central wellbeing components: “feeling useful” was most central in India, while “dealing with problems well” was central in both Israel and the UK. However, the study did not examine differences by age group and was limited to only one measure capturing several psychological wellbeing and affective aspects.

## The Current Study

Despite a growing body of literature applying network analysis to adolescent wellbeing, two main gaps remain. First, few studies have examined the interconnections between subjective wellbeing and psychological wellbeing indicators, and none has used a comprehensive set of subjective wellbeing and psychological wellbeing measures. Second, evidence is lacking on whether the structure of wellbeing networks may differ across crucial developmental stages (e.g., early vs. middle adolescence). This question is particularly salient, given that adolescence is marked by rapid and multidimensional changes in cognitive, emotional, and social functioning. To address these gaps in the literature, the current study aims to ask how different subjective wellbeing and psychological wellbeing components interact in adolescence, and how these network structures vary across the crucial developmental periods of early and middle adolescence (ages 11–14 and 15–18, respectively). By exploring these questions, this study contributes to the understanding of how wellbeing is structured during adolescence, and identifies potential targets for age-sensitive policy and intervention.

## Method

### Sample

Data collection was conducted in May 2024 via an online questionnaire hosted on Qualtrics. Schools were invited to participate based on their affiliation with the International Baccalaureate (IB) school system, as many had previously expressed interest in the IB’s another school project. This provided an opportunity to reach out to IB schools across diverse global regions, though it is recognised that these are not nationally representative samples. All hypotheses, sampling plans, and analytic strategies were pre-registered prior to data analysis at the Open Science Framework (Zhou, 2024). The study received ethical approval from the University of Oxford’s Central University Research Ethics Committee (CUREC; Ethics Approval Reference: R90787/RE001). A passive parental consent (opt-out) procedure was used, and adolescents provided active assent at the beginning of the questionnaire, with the option to withdraw at any time. Students completed the questionnaire during school lesson time. The final sample consisted of 6,445 students aged between 11 and 18 years, drawn from 38 schools across 24 countries (51.9% aged 11–14 and 48.1% aged 15–18). The following table contains information about participant demographics, and country-level representation.

The original English questionnaire was translated into French and Spanish with the support of two native-speaking collaborators. Translation quality was ensured through group discussions involving a subject expert (a postdoctoral-level native speaker), a professional translator (a native speaker), and a researcher for each language. Together, they compared and refined the final versions. Where official translations of the questionnaire were available, these were also reviewed and compared to the team’s versions to enhance consistency and validity. Back-translation was then conducted using ChatGPT-4 to assess semantic accuracy and consistency across language versions. The final translations were confirmed once the back-translations aligned closely with the original English questionnaire. All participants attended schools within the IB system, where English, Spanish, and French are the official languages of instruction. As a result, the survey was administered in these three languages across countries.

### Measures

Participants completed a battery of self-report measures capturing multiple dimensions of subjective wellbeing and psychological wellbeing.

#### Overall life evaluation

This was assessed using three single-item measures rated on scales from 0 to 10: Overall Life Satisfaction, Current Life Evaluation, and Future Life Evaluation. The latter two were adapted from the Cantril Ladder (Cantril, 1965), a widely used tool in global wellbeing research that asks respondents to rate their life on a “ladder” from the worst to the best possible life.

#### Domain-specific life satisfaction

This was measured with 14 items, also using 0 to 10 scales, covering satisfaction across a range of life domains including relationships (e.g., family, friends, teachers), environment (e.g., home area, safety), self-perception (e.g., appearance, health, future expectations), and daily life (e.g., learning at school, time use). These items were adapted from previous large-scale international studies of child and adolescent wellbeing (e.g., Casas et al., 2012).

#### Satisfaction With Life Scale

The Satisfaction with Life Scale (SWLS; Diener et al., 1985) consists of 5 items rated on 7-point Likert scales ranging from Strongly disagree to Strongly agree. It captures global cognitive evaluations of life, such as “I’m satisfied with my life.”

#### Scale of Positive and Negative Experience

Affective wellbeing was assessed using 12 items from the Scale of Positive and Negative Experience (SPANE; Diener et al., 2010). This includes 6 items for positive affect (e.g., joyful, contented) and 6 items for negative affect (e.g., sad, afraid), each rated on 5-point scales from ‘Very rarely or never’ to ‘Very often or always’, referring to the past four weeks.

#### Flourishing Scale

The Flourishing Scale (Diener et al., 2010) includes 8 items assessing aspects such as life purpose, competence, virtue, and social contribution, using 7-point Likert scales from Strongly disagree to Strongly agree.

#### Short Warwick-Edinburgh Mental Well-Being Scale

The Short Warwick-Edinburgh Mental Well-Being Scale (SWEMWBS; Stewart-Brown et al., 2009; Tennant et al., 2007) includes 7 items measuring positive affect and psychological functioning (e.g., optimism, problem-solving, feeling connected), rated on 5-point scales from ‘None of the time’ to ‘All of the time’, referring to the past two weeks.

#### Age-group classification

The study followed a broader definition of adolescence (ages 10–24; Sawyer et al., 2018) and further categorized the sample into early and middle adolescence to facilitate comparability with recent research and to reflect extended developmental transitions. However, the ongoing debate about the boundaries of adolescence is acknowledged, and the study recognizes that such categorizations may not fully capture developmental or contextual variation.

### Network Analysis Procedures

This study followed established reporting standards for psychological network analysis and relevant methodological guidelines (Burger et al., 2022; Epskamp et al., 2018). Network analysis was conducted in R (version 4.4.2) in March, 2025.

The following process has been meticulously replicated across the entire sample as well as in the various age groups. These subgroups include (a) those within the age range of 11 to 14 (referred to as early adolescence), and (b) those within the age range of 15 to 18 (referred to as middle adolescence).

#### Preliminary analysis: assessing topological overlap and multicollinearity

To ensure the validity of the network structure, the study first assessed potential topological overlap, which can result from excessive conceptual similarity between items and inflate node strength estimates. Unique Variable Analysis (UVA) was conducted using the EGAnet package (version 2.1.0) in R (version 4.4.2), applying a stringent cut-off of 0.3 to retain only variables with distinct contributions (Hair et al., 2019). In addition, multicollinearity was evaluated by calculating Variance Inflation Factors (VIFs) for each variable, ensuring that all values remained below the commonly accepted threshold of 5, thereby minimizing redundancy and enhancing the interpretability of the network (James et al., 2013).

#### Network estimation

To ensure that the network analysis captured only unique associations between variables while accounting for potential confounds, both categorical (school, language used, countries and gender) and numerical variables (age) prior to computing partial correlations were controlled for. This approach mitigates potential biases due to uneven group sizes across countries or language groups. The network structure was estimated using the qgraph (version 1.9.8) and bootnet (version 1.6) packages (Epskamp et al., 2018). Pairwise associations between items, conditioned on all other items, were computed using the cor_auto function in qgraph. To derive a clear psychometric network model, the graphical Least Absolute Shrinkage and Selection Operator (gLASSO) was applied with the Extended Bayesian Information Criterion (EBIC) set at γ = 0.5, and a minimal lambda ratio of 0.1 (Epskamp et al., 2018). This approach selects the most robust edges while minimizing false positives. Network visualization were conducted using spring algorithm of qgraph R package, and the node colour were added through the Inkscape app for a clearer visual presentation. The study also reports network density, edge weights and standardized centrality indices, mainly node strength.

#### Network accuracy and stability

The network accuracy and stability were assessed using the bootnet R package (version 1.6). To assess the accuracy of edge weights, nonparametric bootstrapping with 10,000 resamples was performed, which involves repeatedly estimating the network on resampled datasets and computing the variability of edge weights (Efron, 1979). The study also plots the bootstrapped confidence intervals (CIs) for estimated edge parameters. A wide bootstrapped CIs indicating a less accuracy to interpret the strength of an edge (Epskamp et al., 2018).To evaluate the stability of node strength and edge weights, a case-dropping bootstrap procedure with 10,000 resamples was conducted, using a network based on a Spearman correlation matrix. Correlation stability (CS) coefficients was reported, which indicate maximum drop proportions to retain correlation of 0.7 in at least 95% of the samples between the full sample’s estimates and those from the bootstrapped samples. In this case, stability indices should exceed 0.25, and preferably 0.5 (Epskamp et al., 2018).

Finally, to compare the differences in edge weights or centrality, bootstrapped difference tests (with 95% CIs) were carried out using non-parametric bootstrap results (Epskamp et al., 2018). Centrality (strength) was also plotted to assess whether the strengths were significantly different from each other.

#### Network comparison test

Network Comparison Test (NCT) was conducted to examine differences in the psychological networks of early (ages 11–14) and late (ages 15–18) adolescence. Using the NCT() function from the EGAnet package in R, differences in overall network structure, global connectivity strength, and specific edge relationships between psychological variables were tested. The analysis included 5,000 permutations to ensure stable statistical inference. Significant differences in network structure (M-statistic, p-value) and global strength (S-statistic, p-value) were tested. Additionally, specific psychological connections that differed significantly across age groups and examined shifts in centrality measures (i.e., strength, betweenness, and closeness) were identified, to determine if any psychological factors became more or less influential in late adolescence. To control for multiple comparisons, False Discovery Rate (FDR) correction was applied. Visualization of the results was conducted to illustrate significant edge and centrality differences between the two networks.

## Results

### Preliminary Analysis

The final sample consisted of 6,445 students aged between 11 and 18 years, drawn from 38 schools across 24 countries. Prior to analysis, the Interquartile Range (IQR) method was used to identify potential outliers. While all participants fell within the lower bound, a small number exceeded the upper bound. These cases were retained, as further inspection indicated no data quality issues, and their elevated values were likely attributable to contextual factors (e.g., temporary internet delays during submission). Assessment of multicollinearity showed that all Variance Inflation Factors (VIFs) were well below the conventional threshold of 5 (range: 1.2-3.0, see Appendix 1), indicating low redundancy and supporting the robustness of the network estimation. The dataset contained no missing data (understood as all responses collected from all participants). The sample was balanced across age groups, with 51.9% aged 11–14 (early adolescence) and 48.1% aged 15–18 (middle adolescence), although this proportion varied substantially across countries, as shown in Table 1, which presents more details about the sample. In terms of gender, 45.2% identified as boys, 51.7% as girls, and 3.1% as prefer not to say. Survey language distribution was as follows: 82.2% completed the survey in English, 11.7% in French, and 6.2% in Spanish.

**Table 1:** Sample characteristics by country

Country	Respondents	Number of schools	English	Spanish	French	Age 11–14	Age 15–18	Boy	Girl	Prefer not to say gender
Argentina	202	1	1.8%	98.0%	0.0%	56.4%	43.6%	43.6%	56.4%	0.0%
Armenia	135	1	91.1%	3.7%	5.2%	0.0%	100.0%	52.6%	47.4%	0.0%
Austria	72	1	100.0%	0.0%	0.0%	62.5%	37.5%	52.8%	45.8%	1.4%
Bahrain	303	1	100.0%	0.0%	0.0%	66.7%	33.3%	54.8%	42.6%	2.6%
Bangladesh	132	2	100.0%	0.0%	0.0%	79.6%	20.5%	53.0%	44.7%	2.3%
Canada	1202	2	46.5%	0.3%	53.2%	61.8%	38.2%	49.1%	48.0%	2.9%
China	156	1	100.0%	0.0%	0.0%	80.8%	19.2%	48.1%	43.6%	8.3%
Denmark	300	2	98.3%	1.3%	0.3%	30.0%	70.0%	43.3%	54.0%	2.7%
Ecuador	109	1	3.7%	96.3%	0.0%	42.2%	57.8%	48.6%	49.5%	1.8%
Egypt	155	1	89.0%	0.0%	11.0%	51.0%	49.0%	54.8%	45.2%	0.0%
Estonia	172	1	99.6%	0.0%	0.4%	48.9%	51.2%	41.9%	54.1%	4.1%
Germany	437	1	95.6%	3.9%	0.5%	43.0%	57.0%	46.5%	51.5%	2.0%
Ghana	64	1	96.9%	1.6%	1.6%	7.9%	92.1%	58.3%	41.7%	0.0%
India	755	4	100.0%	0.0%	0.0%	75.2%	24.8%	48.9%	50.9%	0.3%
Japan	139	1	99.3%	0.0%	0.7%	75.4%	24.7%	49.2%	50.1%	0.7%
Mexico	98	1	43.9%	56.1%	0.0%	91.8%	8.2%	60.2%	38.8%	1.0%
Russia	145	1	100.0%	0.0%	0.0%	49.7%	50.3%	50.3%	48.3%	1.4%
Sint Maarten	163	1	99.4%	0.0%	0.6%	66.7%	33.3%	41.1%	54.6%	4.3%
Slovakia	82	1	100.0%	0.0%	0.0%	53.7%	46.3%	52.4%	46.3%	1.2%
Sweden	312	2	97.1%	1.6%	1.3%	42.0%	58.0%	44.9%	52.9%	2.2%
The Netherlands	94	1	96.8%	2.1%	1.1%	81.9%	18.1%	47.9%	51.1%	1.1%
Turkey	572	4	100.0%	0.0%	0.0%	4.4%	95.6%	45.3%	52.3%	2.5%
United Arab Emirates	451	2	99.3%	0.2%	0.4%	65.6%	34.4%	46.3%	49.0%	4.7%
United Kingdom	95	2	97.9%	0.0%	2.1%	92.0%	8.0%	27.3%	67.3%	5.3%

### Network Structure and Centrality

**Fig. 1:**
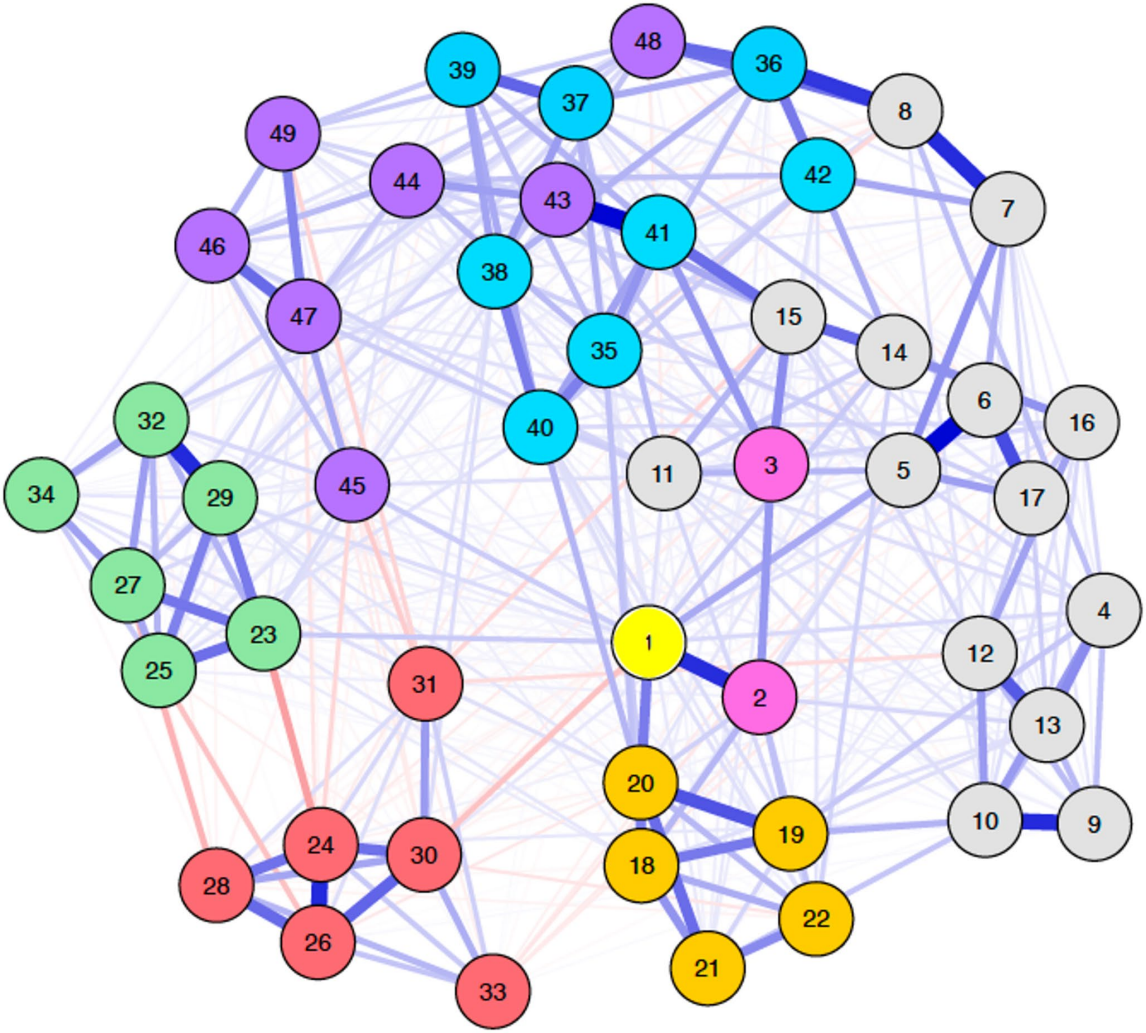
The estimated whole-sample network. Note 1. Overall satisfaction. 2.Cantril Ladder (now). 3.Cantril Ladder (future). 4.The People You Live with. 5.Your Life as Student. 6.Things You Have Learned at School. 7.Other Peers in your Class. 8.Your Friends. 9.The Area where You Live. 10.The Things You Have. 11.How You Use Your time. 12.With your Safety. 13.Your Freedom. 14.The Way You Look. 15.What May Happen To You Later in Life. 16.Your Health. 17.Your Relationships with Teachers. 18.LS1-close To my Ideal. 19.LS2-conditions Are Excellent. 20.LS3-I’m Satisfied with my Life. 21.LS4-gotten Important Things. 22.LS5-change Almost Nothing. 23.Positive. 24.Negative. 25.Good. 26.Bad. 27.Pleasant. 28.Unpleasant. 29.Happy. 30.Sad. 31.Afraid 32.Joyful 33.Angry. 34.Contented. 35.FS1-purposeful and Meaningful Life. 36.FS2-social Relationship. 37.FS3-daily Activities. 38.FS4-contribute To the Wellbeing of Others. 39.FS5-capable in Important Activity. 40.FS6-good Person and Live a Good Life. 41.FS7-optimistic about my Future. 42.FS8- People Respect Me. 43.WEMWBS1-optimistic about the Future. 44.WEMWBS2-useful. 45.WEMWBS3-relaxed. 46.WEMWBS4-deal with Problems Well. 47.WEMWBS5-think Clearly. 48.WEMWBS6-feel Close To Other People. 49.WEMWBS7-make Up my Own Mind.

The estimated whole-sample network exhibited a mean edge weight of 0.0173 (SD = 0.0456) and a density of 0.4804, reflecting a moderately interconnected structure (Fig. 1).

Centrality was evaluated using strength, closeness, and betweenness, with strength forming the basis for the rankings. ‘Overall Satisfaction’, ‘LS3’ (“satisfied with my life”), and ‘Your Life as a Student’ emerged as the most central nodes across all three metrics, which was followed by ‘Optimism about the Future’ (Fig. 1).

**Fig. 2:**
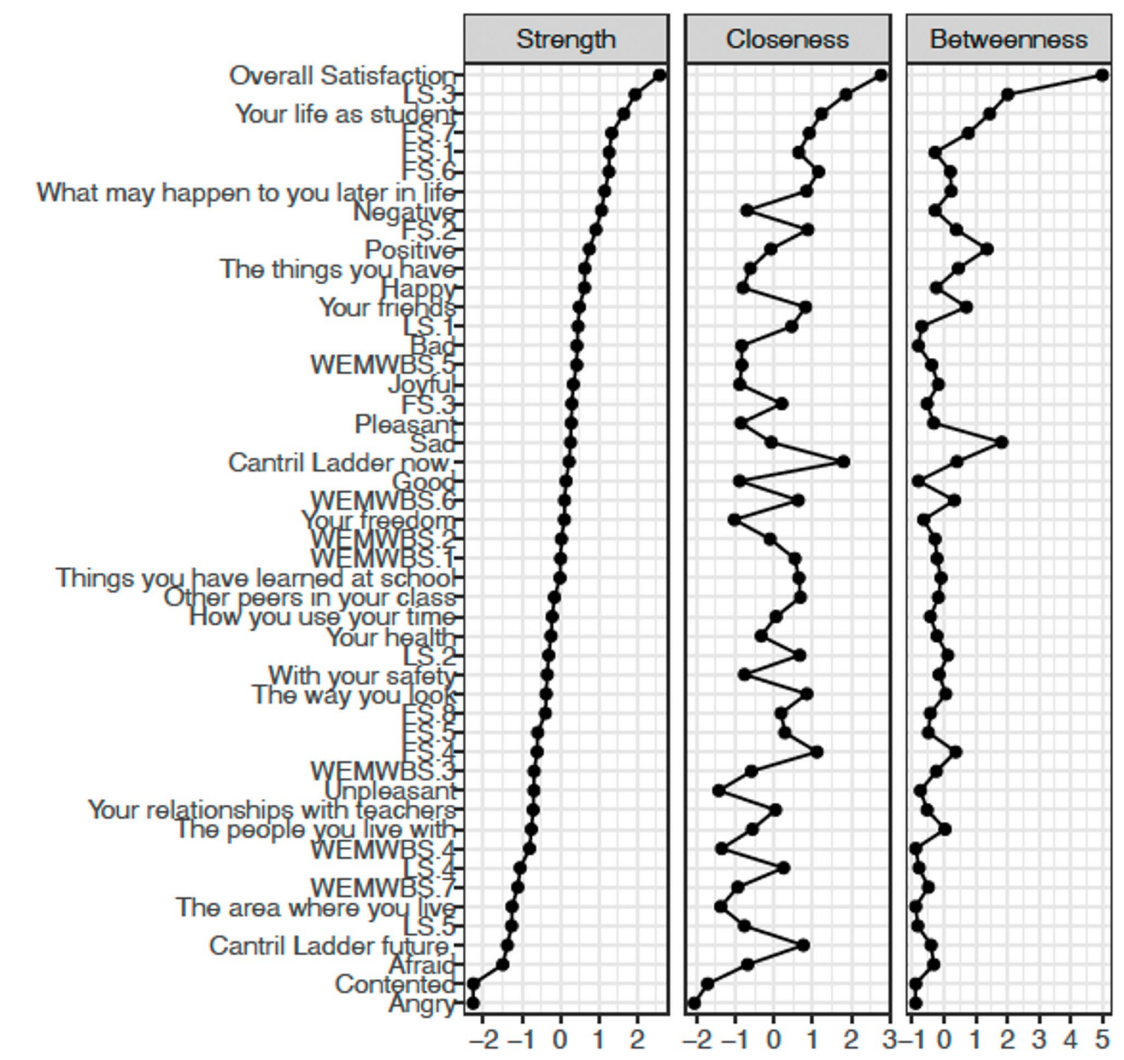
The centrality of estimated whole-sample network

In particular, in terms of strength centrality (Fig. 2), Overall Satisfaction had the highest value (2.58), followed by LS3 (“I’m satisfied with my life”) at 1.95 and Your Life as a Student at 1.65. The measures with the lowest strength centrality were Afraid (-1.50), Contented (-2.26), and Angry (-2.27). For betweenness centrality, Overall Satisfaction again led with a value of 4.99, with LS3 (“I’m satisfied with my life”) at 2.01 and Sad at 1.82. The lowest betweenness values were found for LS5 (“change almost nothing”), The Area Where You Live, and Angry. Closeness centrality was highest for Overall Satisfaction (2.77), followed by LS3 (1.86) and Cantril Ladder (Now) (1.80). WEMWBS-4 (“deal with problems well”), The Area Where You Live, and Unpleasant had the lowest closeness scores.

The strongest positive edges in the network (Fig. 1) were Your Life as a Student – Things You Have Learned at School (0.36), WEMWBS-1 (“optimistic about the future”) – FS7 (“optimistic about my future”) (0.36), Joyful – Happy (0.30), Bad –Negative (0.30), and Other Peers in Your Class – Your Friends (0.30). The strongest negative associations were observed between Negative – Positive (-0.117) and Unpleasant – Pleasant (-0.091).

Metrics of network structure and centrality measures obtained for the early and middle group can be found in Appendix 2.

### Network Accuracy and Stability

The bootstrapped confidence intervals (CIs) of the estimated edge weights revealed narrowed intervals (see Appendix 3), suggesting high accuracy in network estimation. The stability of node strength and edge weights was assessed using a case-dropping bootstrap procedure. The centrality stability (CS) coefficient for node strength and edges was 0.75, exceeding the recommended cutoff of 0.5, indicating a highly stable network. This suggests that up to 75% of the data could be removed while still maintaining a correlation of 0.7 with the original dataset at 95% certainty, further supporting the robustness of the estimated network structure. For both 11–14 and 15–18 age group, the network also demonstrated high stability, with centrality and edge weight CS-coefficients of 0.75, indicating that up to 75% of cases could be dropped while still retaining a correlation of at least 0.7 with the original network in 95% of bootstrap samples.

### Network Comparison Test (NCT) Results

**Fig. 3:**
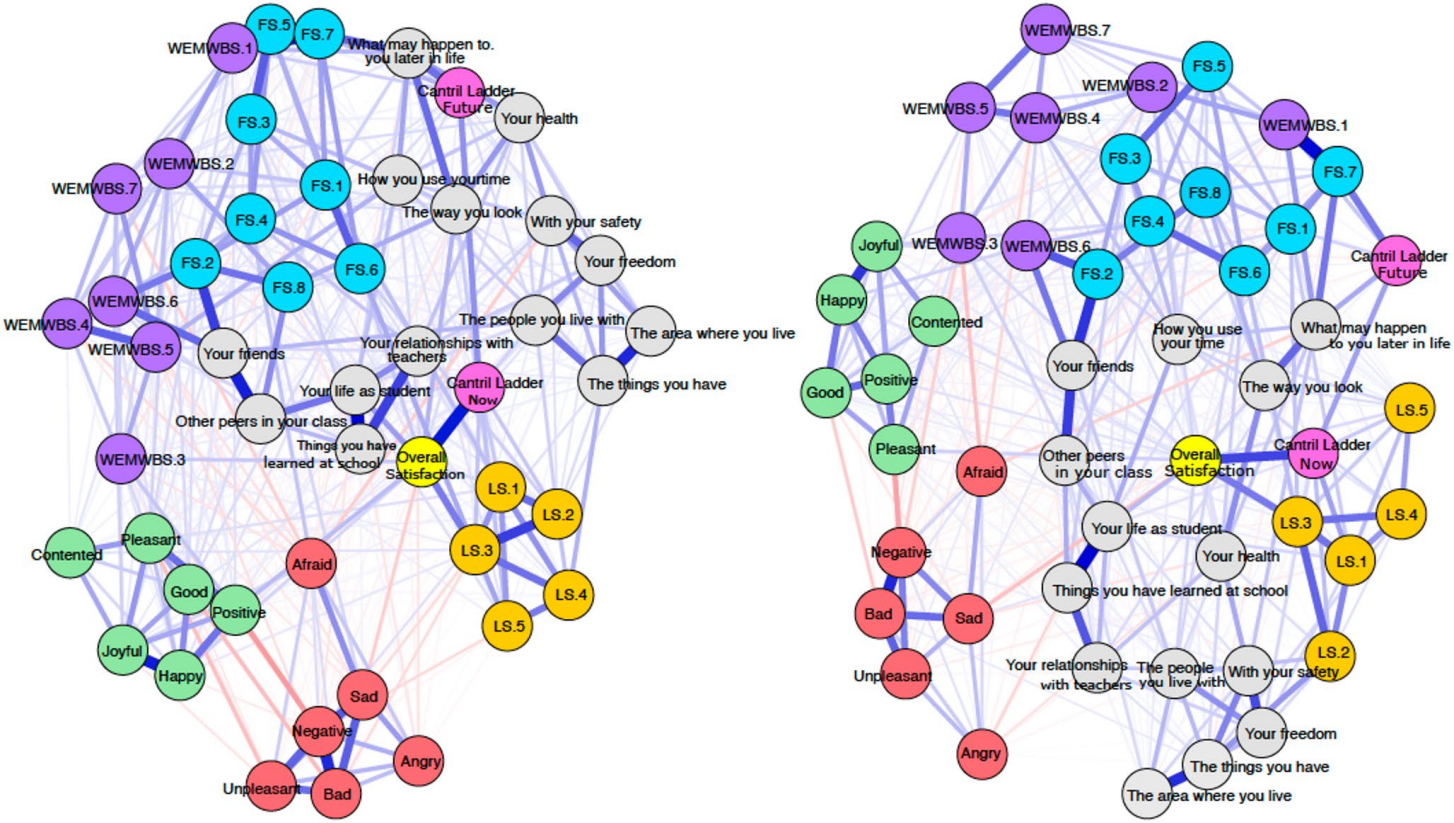
The networks of early adolescents(left) and middle adolescents (right)

Networks were compared for early adolescents (11–14 years) and middle adolescents (15–18 years) using the Network Comparison Test (NCT; van Borkulo et al., 2017) with 5,000 permutations. First, global strength was assessed, finding no significant difference between the two age groups (S = 0.4268, *p* = 0.161), indicating that the overall level of connectivity among wellbeing indicators is comparable in early and middle adolescence (Fig. 3).

Next, the network invariance test revealed a significant difference in overall structure (m = 0.1029, *p* = 0.0249), demonstrating that, despite similar global strength, the specific configuration of connections varies by age. Edge-weight comparisons highlighted several of these age-linked shifts: the association between Overall Life Satisfaction and Pleasant was stronger in the older group (*p* = 0.00020), the link between Your Friends and Your Relationships with Teachers was stronger in the younger group (*p* = 0.00020), and the connection between The Things You Have and LS2 (‘conditions are excellent’) was again stronger in older adolescents (*p* = 0.00040). These differences point to changes in how particular aspects of wellbeing relate to one another across adolescence.

When node centrality was examined, three indicators: Bad, WEMWBS-1 (‘optimistic about the future’), and WEMWBS-3 (‘relaxed’), showed significantly higher strength centrality in the 15-18-year-old network than in the 11-14-year-old network (all *p* < 0.01). This suggests that negative affect and future-orientation and calmness play a more dominant role in the wellbeing network of older adolescents. Conversely, the single-item Cantril Ladder (Now) was significantly more central for younger adolescents (*p* = 0.042), indicating that their momentary global life evaluation is more important in early adolescence.

## Discussion

Despite growing discussion on adolescent wellbeing, significant gaps remain in our understanding of how subjective and psychological wellbeing indicators link and how these wellbeing network structures evolve across different developmental stages. Current literature often lacks the breadth to explore the complex interplay between hedonic and eudaimonic constructs simultaneously, particularly within diverse, multinational contexts. This study applied psychometric network analysis to a large, cross-national sample of 6,445 students (11–18 years) drawn from a globally diverse but non-nationally representative sample of 24 countries, modelling the interplay among 49 indicators of subjective wellbeing (life satisfaction, affect) and psychological wellbeing (flourishing and positive mental functioning). The study revealed that overall life satisfaction and satisfaction with student life consistently remained the most central nodes, and a developmental reorganization occurred from early to middle adolescence; this shift moved from present-oriented evaluations toward future-oriented expectations and regulatory resources.

First, a pooled network was estimated to examine the structure of adolescent wellbeing across the full sample and identify its most central components. Overall life satisfaction, satisfaction with life as a student, and optimism about the future emerged as the most central and interconnected elements within a moderately dense and highly stable network. Then a Network Comparison Test to explore developmental differences between early (ages 11–14) and middle (15–18) adolescence was employed. While the overall strength of connections was similar across age groups, the structure of the network and the centrality of specific nodes, such as negative affect, relaxation, and future optimism, shifted significantly with age. In the sections that follow, these findings are interpreted in light of developmental and wellbeing theory, highlight implications for policy and educational practice, and note limitations and directions for future research.

Across all analyses and all groups (whole sample, age 11–14, age 15–18), Overall Satisfaction consistently emerged as the most central node, across strength, closeness, and betweenness centrality. And similarly, for LS3 (“I’m satisfied with my life”) was among the top hubs. Their high centrality reflects close ties to a range of other wellbeing indicators, suggesting adolescents’ general life satisfaction is tightly interconnected with many other components, serving as a key integrative “hub”. This finding aligns with previous research emphasizing the foundational role of overall life satisfaction in youth wellbeing (OECD, 2021). This study has implications for simple monitoring and confirms a single general life satisfaction item can effectively summarize broad adolescent wellbeing in a multi-country dataset.

Another life satisfaction related item Cantril Ladder (Now) was consistently among the top three nodes across all samples for closeness centrality, but its strength and betweenness centrality was much lower than that of Overall Satisfaction and LS3 (“I’m satisfied with my life”). This suggests while many aspects of wellbeing are efficiently connected to present life evaluation through Cantril ladder, these connections are not as strong or numerous as those anchored by the other two global satisfaction measures (similar result can also be found in Marquez et al., 2025). This may be due to a more abstract and evaluative nature of the Cantril ladder, which ask participants to rank themselves on a hypothetical ladder and is considered to require more abstract, comparative, or externally referenced reasoning than the Overall satisfaction item, which tends to elicit more affective or intuitive appraisals (Fleurbaey & Schwandt, 2015; Nilsson et al., 2024). This may potentially reduce the integration of the Cantril Ladder (now) with other wellbeing indicators.

Although affective experiences are often treated as core elements of wellbeing, the findings reveal distinct roles for individual emotions within the broader adolescent wellbeing network. While negative affect items such as Angry, Contented, Afraid, Unpleasant were consistently peripheral in the network, the item ‘sad’ exhibited a high betweenness centrality. This suggests that sadness plays a bridging or mediating role within the wellbeing structure, even though it is not the most directly connected node. Notably, sadness has been identified as central, alongside other negative moods like anxiety and worry, in a network of Chinese high school students, though their use of a distress-oriented scale and an older, more homogeneous sample (Wang et al., 2023). Together, these findings point out the unique role of sadness in adolescent wellbeing networks across different contexts, emphasizing its importance as both a potential bridge and a target for intervention. By contrast, positive emotions such as happy and joyful, which were central in previous network studies (e.g., Vieta-Piferrer et al., 2024; Blasco-Belled et al., 2024), were less prominent in the analysis. One likely reason is the inclusion of setting-specific indicators, particularly satisfaction with student life, which showed stronger centrality and may displace more general affective states in adolescent populations.

The findings reveal clear age-related differences: wellbeing in early adolescence is anchored in present-focused experiences, particularly school satisfaction, while in middle adolescence it increasingly depends on emotion regulation and future-oriented thinking. Although the overall connectivity of the networks remained stable across age groups echoing prior findings (Blasco-Belled et al., 2024; Vieta-Piferrer et al., 2024), the structure and prominence of specific nodes differ across age groups.

In early adolescence (11–14), life as a student ranked among the most central components of wellbeing, alongside overall life satisfaction and LS3 (“I’m satisfied with my life”), underscoring the importance of day-to-day school experiences during this stage. By middle adolescence (15–18), optimism about my future rose in centrality, signalling a shift toward future-oriented appraisal. This reordering of central nodes reflects growing cognitive capacities for goal-setting, abstract reasoning, and identity exploration (Shulman & Nurmi, 2010). At the same time, emotional regulation became more influential: relaxed mood and low negative affect (e.g., not feeling “bad”) gained strength, while momentary life evaluation (e.g., Cantril Ladder “Now”) declined. These differences suggest that as adolescents mature, sustaining wellbeing may increasingly require managing internal states and maintaining a sense of forward-looking purpose, even under mounting academic and social pressures (Putwain et al., 2021). In this context, feeling relaxed may reflect the absence of stress or anxiety which are unmeasured yet salient factors in middle adolescence that have been shown to shape network structure (Wang et al., 2023). Taken together, these patterns support developmental theory and suggest age-sensitive priorities for intervention: for younger adolescents, enhancing present satisfaction and school engagement; for older adolescents, cultivating optimism and emotional resilience to navigate transitional demands.

Finally, the network also revealed coherent clusters, around academic life, future orientation, positive and negative affect, and social relationships, while showing strong cross-domain linkages that connect subjective wellbeing and psychological wellbeing. For example, overall life satisfaction bridged cognitive evaluations, emotional states, and eudaimonic traits like meaning and purpose. These patterns reinforce the idea that hedonic and eudaimonic wellbeing are not independent domains but form an integrated system during adolescence, as supported by prior network studies (Blasco-Belled et al., 2024). Importantly, the network structure was highly stable, with strong bootstrap metrics and narrow edge confidence intervals, suggesting that similar patterns would likely emerge in comparable samples. The findings build on prior evidence that found stable wellbeing network structures across gender and residential context (Wang et al., 2023). This evidence is extended by demonstrating network stability across a much larger and more diverse sample, spanning 24 countries and covering ages 11 to 18, and by incorporating a broader range of wellbeing indicators.

### Implications for Policy, Practice, and Research

The findings offer clear guidance for how adolescent wellbeing can be measured, supported, and better understood in both policy and applied settings. Most notably, overall life satisfaction emerged as the most central node across all network metrics making it a powerful structural hub. If practical constraints limit the use of longer scales, this single-item 0–10 measure (“Overall, how satisfied are you with your life?”) provides a reliable and informative summary of adolescents’ broader wellbeing.

Beyond this, the strong centrality of school-related satisfaction and student experiences across a diverse international sample highlights their relevance as core monitoring indicators. Policies aiming to improve adolescent wellbeing should therefore invest in both the present-day context by fostering positive school climates and peer relationships and the future-facing capacities adolescents develop, such as optimism, hope, and purpose. Notably, future orientation became more central in mid-adolescence, suggesting that efforts to build goal-setting and resilience skills become increasingly important with age. Moreover, the selection of specific wellbeing measures is not merely a methodological decision but also reflects deeper assumptions about what constitutes healthy adolescent development. Different tools emphasize distinct element, such as affective balance, autonomy, or relational connectedness, and their use can signal varying priorities across policy, cultural, or developmental frameworks. Thus, while core indicators such as life satisfaction and school engagement should anchor national and international wellbeing strategies, flexibility for local adaptation remains essential, given cultural variation in which components matter most (Shukla et al., 2022).

In relation to intervention, strategies should focus on the most central relevant network nodes. For younger adolescents, boosting daily satisfaction (particularly with school and peer relationships) may yield broad improvements across their wellbeing system. For older adolescents, fostering future-oriented thinking, emotional regulation, and stress coping may be more impactful. Targeted interventions could include mentoring programs, goal-setting workshops, or classroom environments designed to build calm and connection. The bridging role of sadness in the network also suggests it may be a key early marker of vulnerability. Addressing sadness through counselling or social-emotional learning could improve not only mood but also broader wellbeing domains due to its linking role.

The findings also highlight measurement considerations. While network diagnostics confirmed no problematic multicollinearity, semantic overlap was observed between items such as “good” and “positive” or “bad” and “negative.” These pairs, while statistically distinct, may represent overlapping constructs that could be refined in future measurement work. Moreover, based on the observations, terms like “contented”, may not resonate with younger respondents and should be replaced with more developmentally appropriate alternatives. These refinements can improve both the accuracy and accessibility of youth wellbeing assessments.

Given the international sample, future work should build on this by conducting culturally grounded network analyses, and aim to expand to nationally representative samples. While the findings were robust across 24 countries, pooled analyses may mask important context-specific differences. Comparative studies, such as those from Children’s Worlds, the Programme for Student Assessment (PISA), and the Health Behaviour in School-aged Children (HBSC), have shown that the timing and pace of adolescent wellbeing changes vary across countries (Marquez et al., 2024); network models could help determine whether the structure of wellbeing shifts in parallel. In addition to cultural variation, longitudinal designs are needed to track how adolescent wellbeing networks evolve over time and to test directional hypotheses, such as whether optimism fosters later life satisfaction or vice versa. Exploring individual-level moderators, including gender, migrant background, neurodiversity, and sexuality, will further illuminate who experiences these shifts most strongly and why. Finally, experience sampling methods, which capture real-time fluctuations in adolescents’ emotions and thoughts, could offer rich insight into the dynamic interplay of wellbeing components in daily life.

### Limitations

Several limitations should be acknowledged when interpreting these findings. First, the sample (large but drawn from International Baccalaureate schools) may not represent national-school populations (SES, school climate, motivation), limiting generalisability; replication in more diverse settings is needed. Second, uneven age distributions across countries mean some age-group differences could reflect cultural sampling imbalances rather than pure age-related effects. Third, all measures were self-reported in a single online session, raising risks of common-method variance and transient mood effects; longitudinal or experience-sampling designs would facilitate causal inference and temporal claims. Fourth, although translations followed standard procedures, measurement invariance across languages was not tested, so differential item functioning could affect pooled estimates. Fifth, the analyses are cross-sectional and conditional on the chosen item set. Centrality and edge estimates can change with different nodes or controls (Bringmann et al., 2019), and recent work flags potential robustness limits in psychological networks (Huth et al., 2025). Thus, replication and longitudinal modelling are required. Finally, the analyses controlled for several covariates but omitted contextual factors (e.g., SES, family functioning, pandemic effects) that might confound specific associations. Future work should address these issues via cross-cultural invariance testing, multi-group/multilevel network models, longitudinal designs, and parsimonious item selection or scale-level networks.

## Conclusion

The current corpus of literature contains limited exploration of how subjective wellbeing and psychological wellbeing indicators interact, and how the structure of wellbeing networks (how these indicators interact) might differ at different stages of adolescent development. By mapping how subjective wellbeing and psychological wellbeing components interact, this study identifies actionable targets for measurement, policy, and intervention, and clarifies priorities for future research. The findings indicate that adolescent wellbeing is best understood as a dynamic, interconnected system rather than a set of isolated traits. Within this system, overall life satisfaction, school satisfaction, and optimism about the future consistently act as central hubs, underscoring the foundational roles of both present experiences and future-oriented appraisals. Although these core links are stable from early to middle adolescence, meaningful age-related shifts were observed: rising centrality of negative affect (e.g., feeling “bad”), emotional calm (e.g., feeling “relaxed”), and future optimism, alongside declining centrality of momentary life evaluation. These patterns argue for age-sensitive monitoring and potentials for targeted supports.

## Appendix

### Appendix 1. VIF for Each Variable

Variable VIF.

0 const 1.000087.

1 Overall Satisfaction 3.023596.

2 Cantril Ladder (now) 2.234814.

3 Cantril Ladder (future) 1.613175.

4 The people you live with 1.583486.

5 Your life as student 2.439353.

6 Things you have learned at school 1.910655.

7 Other peers in your class 1.720235.

8 Your friends 1.796759.

9 The area where you live 1.502358.

10 The things you have 1.738353.

11 How you use your time 1.668891.

12 With your safety 1.598125.

13 Your freedom 1.768268.

14 The way you look 1.740616.

15 What may happen to you later in life 2.192569.

16 Your health 1.672327.

17 Your relationships with teachers 1.625115.

18 LS-1 2.190502.

19 LS-2 2.076604.

20 LS-3 2.885477.

21 LS-4 1.742763.

22 LS-5 1.588369.

23 Positive 2.312418.

24 Negative 2.100740.

25 Good 2.007192.

26 Bad 2.043766.

27 Pleasant 1.914027.

28 Unpleasant 1.662810.

29 Happy 2.228420.

30 Sad 1.761135.

31 Afraid 1.227960.

32 Joyful 1.960906.

33 Angry 1.247452.

34 Contented 1.317176.

35 FS-1 2.270227.

36 FS-2 2.100896.

37 FS-3 1.906362.

38 FS-4 1.534580.

39 FS-5 1.684989.

40 FS-6 2.225370.

41 FS-7 2.425218.

42 FS-8 1.696875.

43 WEMWBS-1 1.944100.

44 WEMWBS-2 1.756028.

45 WEMWBS-3 1.492478.

46 WEMWBS-4 1.469470.

47 WEMWBS-5 1.684842.

48 WEMWBS-6 1.674953.

49 WEMWBS-7 1.449779.

### Appendix 2. Network Stability and Centrality Results by Age Group

**Ages 11–14**

Sampling levels tested:

- nPerson range: 836 (75% drop) to 3,176 (5% drop).

**Network stability**:

- The centrality stability (CS) coefficient for both edge weights and node strength was 0.75, exceeding the recommended minimum of 0.5. This indicates that up to 75% of cases could be dropped while retaining a correlation of at least 0.7 with the original network in 95% of bootstrap samples, supporting strong network robustness.

**Strongest edges**:

- *Your Life as a Student* – *Things You Have Learned at School*: 0.337.
- *Cantril Ladder (Now)* – *Overall Satisfaction*: 0.305.
- *WEMWBS-1 (“optimistic about the future”)* – *FS7 (“optimistic about my future”)*: 0.334.

**Strongest negative edges**:

- *Negative* – *Positive*: − 0.113.
- *Bad* – *Good*: − 0.074.
- *Pleasant* – *Unpleasant*: − 0.071.

**Node centrality**:

- **Strength centrality**: Highest for *Overall Satisfaction*, *LS3 (“satisfied with my life”)*, and *Your Life as a Student*; lowest for *Contented*, *Angry*, and *The Area Where You Live*.
- **Betweenness centrality**: Highest for *Overall Satisfaction* (4.99), *Positive*, and *LS3* (2.01); lowest for *Contented*, *Angry*, and *The Area Where You Live*.
- **Closeness centrality**: Highest for *Overall Satisfaction*, *Cantril Ladder (Now)*, and *LS3*; lowest for *Contented*, *Angry*, and *Unpleasant*.

These results highlight the central importance of life satisfaction and school experience in the wellbeing network of younger adolescents.

**Ages 15–18**

Sampling levels tested:

- nPerson range: 776 (75% drop) to 2,947 (5% drop).

**Network stability**:

- The CS coefficient for both edge weights and node strength was 0.75, indicating robust stability as in the younger group.

**Strongest edges**:

- *Your Life as a Student* – *Things You Have Learned at School*: 0.389.
- *WEMWBS-1* – *FS7*: 0.385.
- *Bad* – *Negative*: 0.325.

**Strongest negative edges**:

- *Negative* – *Positive*: − 0.120.
- *Afraid* – *WEMWBS-3 (“relaxed”)*: − 0.080.
- *Pleasant* – *Unpleasant*: − 0.106.

**Node centrality**:

- **Strength centrality**: Highest for *Overall Satisfaction*, *LS3 (“satisfied with my life”)*, and *FS7 (“optimistic about my future”)*; lowest for *Angry*, *Contented*, and *Afraid*.
- **Betweenness centrality**: Highest for *Overall Satisfaction*, *LS3*, and *Sad*; lowest for *Contented*, *LS5 (“change almost nothing”)*, and *The Area Where You Live*.
- **Closeness centrality**: Highest for *Overall Satisfaction*, *LS3*, and *Cantril Ladder (Now)*; lowest for *Contented*, *Angry*, and *Unpleasant*.

### Appendix 3. bootstrap of CIs of estimated edge



## Declarations

### Data Sharing Declaration

Analytic code and study materials are available in an OSF repository for review at [https://osf.io/q7pky/overview?view_only=aa95808c3ebd465fae111bcbd93dd867]. Due to multi-country child-data regulations, consent limits, and small-cell disclosure risks, individual-level data cannot be made public. The repository will be made public upon publication.
